# Understanding University Students' Perspectives towards Digital Tools for Mental Health Support: A Cross-country Study

**DOI:** 10.2174/0117450179271467231231060255

**Published:** 2024-02-21

**Authors:** Ilaria Riboldi, Angela Calabrese, Susanna Piacenti, Chiara Alessandra Capogrosso, Susanna Lucini Paioni, Francesco Bartoli, Giuseppe Carrà, Jo Armes, Cath Taylor, Cristina Crocamo

**Affiliations:** 1 Department of Medicine and Surgery, University of Milano-Bicocca, Via Cadore 48, Monza 20900, Italy; 2 Division of Psychiatry, University College London, Maple House 149, London W1T 7BN, UK; 3 Faculty of Health and Medical Sciences, School of Health and Sciences, University of Surrey, Stag Hill, Guildford GU2 7XH, UK

**Keywords:** Digital technology, Mental health, Qualitative research, Students, Universities

## Abstract

**Background:**

Organisational and individual barriers often prevent university students from seeking mental health support. Digital technologies are recognised as effective in managing psychological distress and as a source of health-related information, thus representing useful options to address mental health needs in terms of accessibility and cost-effectiveness. However, university students' experiences and perspectives towards such interventions are little known.

**Objectives:**

We thus aimed to expand the existing base of scientific knowledge, focusing on this special population.

**Methods:**

Data were from the qualitative component of “the CAMPUS study”, longitudinally assessing the mental health of students at the University of Milano-Bicocca (Italy) and the University of Surrey (UK). We conducted in-depth interviews and thematically analysed the transcripts using the framework approach.

**Results:**

An explanatory model was derived from five themes identified across 33 interviews (15 for Italy, 18 for the UK). Students perceived that social media, apps, and podcasts could deliver relevant mental health content, ranging from primary to tertiary prevention. Wide availability and anonymity were perceived as advantages that make tools suitable for preventive interventions, to reduce mental health stigma, and as an extension of standard treatment. These goals can be hindered by disadvantages, namely lower efficacy compared to face-to-face contact, lack of personalisation, and problematic engagement. Individual and cultural specificities might influence awareness and perspectives on the use of digital technologies for mental health support.

**Conclusion:**

Although considering some specific features, digital tools could be a useful instrument to support the mental health needs of students. Since personal contact remains crucial, digital tools should be integrated with face-to-face interventions through a multi-modal approach.

## 1 INTRODUCTION

The poor mental health of university students is a growing concern for public health [1]. Indeed, higher education settings are likely to exacerbate students' vulnerability to psychological distress, especially during the first years of the educational path [2, 3]. Academic challenges and major changes in lifestyle habits often lead to increased social, performance, and financial demands [4].

An increasing trend for common mental health disorders among university students has been highlighted in recent decades, with anxiety and depressive symptoms identified as the most frequent conditions in terms of both incidence and severity [5-7]. Therefore, several educational institutions have prioritised preventative approaches through the provision of well-being centres, psychoeducational programmes, and digital interventions [8-10]. Notwithstanding the efforts made by academic institutions to deploy more structured strategies for common mental disorders among young adults [11, 12], organisational and individual barriers, namely stigma, difficult accessibility, as well as a lack of adequate information about provision, may hinder students in need from seeking psychological assistance [13, 14]. Digital technologies have been demonstrated to provide a concrete opportunity in order to address health challenges [15]. More in detail, digital mental health technologies, defined as any mental health support that is delivered via web-based or mobile-based platforms [16, 17], have been widely recognised as a potential solution to mitigate some of these barriers [18, 19]. Digital mental health tools include successfully virtual-adapted psychological and psychotherapy interventions, which have been reported as effective in the management of depressive and anxiety symptoms, as well as insomnia, alcohol misuse and eating disorders amongst university students [10, 20-23]. Moreover, technology-based instruments could be used for psychological distress relief and as a source of health-related information, mainly through social media, thus potentially ranging from primary to tertiary prevention [24-27]. In addition, the spread of these virtual approaches to mental health, which seems to be a natural trend in the progressive digitalisation of modern society, was further accelerated by social distancing imposed during the COVID-19 pandemic [28, 29], with young people representing the most suitable users, due to their familiarity and extensive exposure to digital communications [17, 30]. Several specific advantages have been identified for digital tools, which university students described as more accessible and cost-effective than solely in-person approaches, as well as being highly flexible [17, 31]. Notwithstanding these benefits, research has identified some disadvantages, namely concerns about confidentiality and the quality of tailored interventions [32, 33]. All these features must be considered in the design process of digital tools to provide appropriate content through congruous devices to support students' mental health [34-37].

Although students' perspectives on the topic have been previously investigated in some specific National contexts [32, 38], the actual use of digital tools for mental health support, along with their relevance in students' lives, may be influenced by cultural specificities, as well as lifestyle habits [39-41]. Diverse strategies for promoting engagement and individual differences could influence the awareness and exploitation of these digital tools [42, 43]. Thus, we carried out in-depth interviews to explore students' experiences, feelings, and perspectives about using digital tools for mental health support in university settings, including cross-cultural comparisons between Italy and the UK.

## 2 MATERIALS AND METHODS

### 2.1 Study Design and Setting

This study was reported in line with the COnsolidated criteria for REporting Qualitative research (COREQ) [44]. Data were derived from the qualitative component of “Caring and Assessing Mental Health of Student Populations at Unimib and uniSurrey: the CAMPUS study”, a large ongoing project longitudinally assessing the mental health of university students enrolled at the University of Milano-Bicocca (Unimib, Milan, Italy) and the University of Surrey (UoS, Guildford, United Kingdom) [45]. Semi-structured in-depth interviews were used to explore students' experiences, feelings and perspectives on the use of digital tools for mental health support. In line with established research, we estimated we would need to conduct 10-20 interviews in order to achieve data saturation [46, 47]. Existing literature on student mental health informed interview guide development (Table **S1**). For the purpose of this paper, we present findings from the interviews that focused on issues related to digital interventions. A University Steering Committee was established to provide advice and promote the project. Interviews were scheduled via the Microsoft Teams online platform by a researcher (I.R.) between September 2021 and April 2022. They were audio-recorded and transcribed verbatim (A.C., C.A.C., S.L.P., S.P.) and lasted 32 min on average.

### 2.2 Participants

Participants were eligible to participate if they were 18 years of age or older and currently enrolled at one of the two universities. They were purposively sampled according to gender and age distribution, nationality, programme of study, and living accommodation to provide a variety of perspectives and experiences. Students meeting the inclusion criteria were approached by key site contacts *via* email and invited to participate in the study. The resulting response rate of nearly 20% met our planned sample size.

### 2.3 Data Analyses

Interview data were analysed thematically [48, 49] using the framework approach [50]. The thematic analysis followed these steps: 1. familiarising with data; 2. generating initial codes; 3. constructing themes; 4. reviewing potential themes; 5. defining and naming themes. The analysis took an exploratory, inductive approach to capture students' experiences and attitudes about digital tools for mental health support. One researcher (I.R.) first read each transcript independently, highlighting initial themes emerging from the data. Then, codes were organised into higher-order themes that represented relevant content of students' perspectives. Themes and their inter-relationships were reviewed by C.T. and J.A. separately, and any differences in the categorisation of data into themes were discussed, leading to the refinement of the original codes. A hierarchical thematic framework emerged as data analysis progressed. Data were then extracted into matrices following a structured approach to facilitate the identification of patterns within and between different groups of students (*e.g*., Unimib *vs*. UoS; male *vs*. female; living at home *vs*. living in university accommodation). The framework matrices were reviewed independently (by I.R., J.A., C.T.) and then through a panel discussion to build a consensus on emergent patterns. In addition, standard descriptive analyses were carried out to summarise the characteristics of participants.

## 3 RESULTS

### 3.1 Sample Characteristics

We conducted a total of 33 individual interviews: 15 involving students from Italy and 18 with students from the UK, of which a total of 26 (79%) were female. The sample characteristics are reported in Table **1**.

### 3.2 Themes and Sub-themes

Five themes were identified from the analysis of transcripts, including i) preferred mode of mental health support delivery, ii) useful content, iii) perceived advantages of digital tools, iv) perceived disadvantages of digital tools, and v) putative roles of digital tools for mental health support. Themes were further categorised into sub-themes, ordered in the text according to their frequency across the transcripts (from most to least frequent). Mental health digital users did not explore additional themes and sub-themes as compared to non-users (Table **1**). For a complete set of participants' quotes, (Table **S2**). The relationships within and between themes and sub-themes were explored, leading to the explanatory model. This framework explained the relationship between mental health needs identified by students, namely the need for accessibility, prevention and psychological support, stigma reduction, as well as personal interaction, and through which mode and potential mechanisms these needs may be met by digital solutions, promoting both primary, secondary and tertiary prevention. The model is shown in Fig (**1**).

#### 3.2.1 Preferred Mode of Mental Health Support Delivery

##### 3.2.1.1 Social Media

Eleven interviewees (33%), equally distributed between UoS and Unimib, identified social media as their preferred mode of mental health support delivery. Younger students, in particular, were most likely to state that this preference was influenced by the wide diffusion of social media, especially among the most recent generations.

“You know, it's like everything is on social media, and it makes it so much easier to reach out to people, especially the youngest ones.” UoS, F.

Instagram^®^ was almost unanimously described as the best option, as it is widespread, direct, easy to access and allows emotional connection among users.

“Probably Instagram is the best social media to use for mental health support because that's what most students use, and it is very direct and easy to access.” UoS, F.

“We are always connected with others on Instagram, and we can share our experiences and emotions in every moment.” Unimib, F.

Some students reported the potential opportunity to share information on mental health via social media and how some of these tools already focused on psychological content.

“I know about some Instagram pages already offering content and articles about mental health.” Unimib, F.

Finally, social media could help decrease the individual perception of being misunderstood or judged about mental health issues.

“Let's say online stuff, especially social media like Facebook, just give young people an insight that they're not alone nor judged.” UoS, F.

“There is an increase of people on social media with real mental health problems talking about their experiences. It helps you to feel understood.” UoS, F.

##### 3.2.1.2 Mobile Apps/Online platforms

Despite the dominance of social media, mobile apps, and online platforms were also mentioned as relevant devices for mental health support, particularly among UK students (mainly mature student participants). Some of the interviewees specifically referred to mobile apps that could potentially disseminate information on mental health, promoting prevention, especially among people who suffer from social anxiety or prefer to remain anonymous.

“On apps, people can just anonymously say what they're going through.” UoS, F.

“I think that an app could be more useful for prevention, in particular for people that suffer from social anxiety.” UoS, M.

Digital apps were also seen as an instrument for receiving direct support and attending psychological sessions.

“I am thinking about an app where people can do some type of psychotherapy or mindfulness.” UoS, F.

“I know there is a very useful app to help students manage their self-harm.” UoS, F.

Finally, online platforms, namely websites and blogs, were considered a potential option for providing mental health support by two students from Unimib, mainly because general knowledge about mental health often relies on online browsing.

“Students are made aware of many issues through almost exclusively the use of Information Technology (IT) means, social media, online pages.” Unimib, F.

##### 3.2.1.3 Podcasts

University students categorised podcasts as a tool that could be potentially employed in mental health support, although to a lesser extent than social media and mobile apps. Four interviewees, mainly from UoS and enrolled in scientific programmes, expressed their opinion about the opportunity to listen to psychological content or even described their personal experience with podcasts focusing on mental health.

“Podcasts about mental health helped me a lot. They didn't change my mood, but they reassured me that I'm not the only one.” UoS, F.

“I would like to listen to something like a podcast on mental health.” Unimib, F.

#### 3.2.2 Useful Content

##### 3.2.2.1 Primary Prevention Content: Psychoeducation/Information on Mental Health

Fourteen (42%) students identified psychoeducation as relevant content to exploit in digital tools for mental health support in order to promote symptoms' normalisation. On the one hand, most respondents generically referred to “*information or advertisement on mental health*”.

“I propose a mobile app where you can log in and find information about mental health.” UoS, M.

“…just general advertisement on mental health through apps.” UoS, F.

On the other hand, information on mental health was explicitly defined by other interviewees as motivational messages, as well as professionals' opinions on the topic, together with practical advice on how to search for help.

“I think motivational quotes could be useful to increase awareness about mental health.” UoS, F.

“On Instagram I follow many psychologists who do a lot of awareness about mental health in a professional way.” Unimib, F.

“Digital tools can allow you to know how to search for a psychologist.” Unimib, F.

One of the students from Italy highlighted the importance of sharing information based on scientific data alongside motivational materials.

“You can use time to read sentences on social media, not only motivational, but with scientific data on mental health.” Unimib, F.

Finally, one of the participants from UoS specified that an institutional account could be the best option to reach the largest number of students.

“...a university account with information on mental health. University would need to advertise it in order to reach as many people as possible.” UoS, F.

##### 3.2.2.2 Tertiary Prevention Content: Psychological Support/Psychotherapy

Digital tools could also represent a means to offer direct support to young people who already struggle with psychological symptoms. Only UK participants, mainly the mature ones who were enrolled in scientific programmes, described psychological support as a variety of actions ranging from “*talking and asking questions about your mental health*” to proper psychotherapy sessions.

“I am thinking about something simple with a space where you can ask questions and [receive] support from someone who is a real person.” UoS, M.

“It could be useful to create an app to connect you with your tutors or the university's Centre of Wellbeing in a more fluid way to receive general support.” UoS, M.

“…an app where people can receive some type of psychotherapy.” UoS, F.

##### 3.2.2.3 Secondary Prevention Content

###### 3.2.2.3.1 Peer Support

Peer support is defined as a form of social-emotional support offered by an individual with a shared lived experience [51]. In our sample, especially according to the UK students, including international ones, testimonials about personal experiences on psychological issues were considered useful for providing mental health support. This type of content is regarded as helpful for people at risk of developing psychological symptoms or as a first approach in an early phase of a poor mental health condition.

“I feel like social media could be a way of sharing personal stories, and it could be really good for peer support on mental health.” UoS, F.

“I propose to create and share videos, interviews, experiences, and personal testimonials precisely referred to the aspect of the student's mental health.” Unimib, F.

Some students elaborated by saying peer support could be an instrument to increase awareness by sharing advice and coping strategies.

“There is an increase of people talking about their experiences with mental health problems. It could be useful for advice and real coping mechanisms.” UoS, F.

###### 3.2.2.3.2 Specific Interactive Content

Specific interactive content, namely tools to track emotions, monitor mood and schedule healthy daily activities, were discussed by some female and male students as a way to help with early detection and support of psychological symptoms. Moreover, active interaction was considered an essential aspect of successful digital tools expected to involve a large number of users.

“I propose an app that gives people small activities to do throughout the day and a tracking of their emotions. At the end of the day, they have to report back what they did and how they feel, and they receive some advice.” UoS, F.

“Digital interventions need to have interactive content, like a tracking of the emotions to detect early mental health problem.” Unimib, M.

#### 3.2.3 Perceived Advantages of Digital Tools

##### 3.2.3.1 Availability

Digital tools were described by younger students as highly available, primarily because they are easily accessible as a whole and in comparison to face-to-face services.

“Booking an appointment to go physically to a psychological session is harder than just using a digital platform, which is much more accessible.” UoS, F.

“I think digital tools help, especially with students. I think accessibility is very important, as students are, on the whole, quite lazy.” UoS, M.

Moreover, in the interviewees' opinion, the wide diffusion of digital tools, above all social media, and the “*everything is online*” reality guaranteed being able to reach a large pool of university students.

“I think it is really good [to use digital tools for mental health] since so many people use their phones and they are always online.” UoS, F.

##### 3.2.3.2 Anonymity

Besides availability, the opportunity to remain anonymous was raised as one of the main advantages of digital tools, mainly by students from the UK enrolled in scientific degree courses. The chance to seek help without attracting attention may be a good way to approach mental health support since, in students' opinion, personal contact might be stressful or even a source of social anxiety.

“Some people, including me, may not want to go and speak to someone face-to-face because it is a bit stressful, and it's harder than speaking over the computer or something digital.” UoS, F.

“I think making a phone call to a helpline is much more difficult for students than using an app and doing things behind the screen. They can have some social anxiety and prefer to be anonymous.” UoS, M.

Moreover, a student from Italy recognised the anonymity of digital tools as a way to overcome the embarrassment still associated with discussing mental health issues.

“Maybe someone is embarrassed to say that he has symptoms. Digital tools could be useful because they are anonymous.” Unimib, F.

#### 3.2.4 Perceived Disadvantages of Digital Tools

##### 3.2.4.1 Lower Efficacy than Personal Contact

Although personal contact was defined as potentially stressful and a possible source of social anxiety, the most common disadvantage of digital tools described by students was their lower efficacy as compared to face-to-face interaction. Younger female students, mainly from the UK, expressed their opinions or personal experiences about the superiority of in-person relationships compared to those mediated via a screen, mainly in the context of treatment interventions.

“I think they (digital tools) can only be used together with, not instead of, personal contact. I prefer personal contact.” UoS, F.

“Honestly (digital for mental health) is much less effective and attracts much less than an in-person one-to-one meeting.” Unimib, F.

This common belief was potentially exacerbated by the COVID-19 pandemic, during which virtual lectures were mandatory, and there was a general increase in time spent online. According to the student's quote below, remote instruments should not be used for mental health support nor as the sole instrument of teaching.

“Digital doesn't make much sense. I don't think it could be useful for mental health because, in my opinion, human relationships are essential. Physical contact has a completely different impact on mental health, and I realised it during the pandemic. Online stuff can be useful but not for teaching nor for mental health support.” Unimib, M.

Other students highlighted the seriousness of mental health issues, described as “*a real-world problem*” and deserving not a digital but a “*real-world solution*”, mainly for more severe conditions.

“Mental health issues are a real-world problem, and you have to address them in the real world. I think you can simply search for National Health Service. I don't trust internet stuff.” UoS, M.

“When you have a serious mental health problem, it is better to go to speak to someone face-to-face.” UoS, M.

One of the students from Italy described digital interactions as less attractive than human contact, especially when a habit of use was not yet established.

“I would honestly see [digital tools] as less attractive than human contact, especially at the beginning.” Unimib, F.

Finally, the negative aspect of digital tools, namely social media, was raised by one of the interviewees.

“I felt more the stressful aspect of social media instead of the beneficial one. Unhealthy competition and comparison with others. Personal contacts are better.” UoS, M.

##### 3.2.4.2 Lack of Personalisation

Another disadvantage outlined by older students from the UK was the lower personalisation as compared to in-person interactions, which could potentially impact digital technologies. In the participants' opinion, psychological issues were considered complex conditions barely standardisable. Therefore, tailored content was seen as a crucial feature of successful preventive and therapeutic interventions, though not always achievable.

“For university students, a digital intervention needs to be more personalised than the ones that you can find nowadays.” UoS, M.

“Sometimes [digital tools] cannot be specific enough for all types of mental health problems.” UoS, M.

##### 3.2.4.3 Problematic Engagement

Both students from UoS and Unimib focused on the challenge of developing a lasting habit in the use of digital tools for mental health support, especially if mobile apps were considered.

“I think the main sort of thing to worry about in the use of digital tools for mental health support is how to get people to use them over time.” UoS, F.

One of the students called for more engaging digital content so as to be able to compete with face-to-face contact.

“I would honestly see [digital tools] as less attractive than human contact. Users need to see that instead, digital applications could be interesting to continue to use them.” Unimib, F.

#### 3.2.5 Putative Roles in Mental Health Support

##### 3.2.5.1 Complement/Extension of Standard Treatment

Interviewees defined digital tools as a possible complement to standard treatment. Students from both universities, with a predominance of UoS students, stated that digital instruments could be a good option as an initial way to become familiar with mental health settings.

“Digital platforms are very useful as a starting point for mental health support. Then you can go on together with face-to-face psychotherapy.” UoS, F.

“I know apps about mental health, and I think they could be a first aid in mental health support.” Unimib, F.

Otherwise, two students recognised the utility of digital tools not only in the first phase of the treatment but also as support in the context of a multi-modal programme.

“I think [digital tools] would be part of a multi-pronged programme, also including personal contact.” UoS, F.

“I do not believe so much in digital tools as a sole support for mental health. Maybe they can be of help during therapy.” Unimib, F.

Finally, digital interventions were considered an appropriate mental health support strategy, but only for less severe conditions.

“I think digital utility depends on the severity of the mental health problem. It is really useful for initial situations.” UoS, M.

“For students suffering from a very superficial and initial problem, they [digital tools] can also be helpful, but not as the unique treatment.” Unimib, F.

##### 3.2.5.2 Prevention

The other specific role assigned to digital tools by younger participants was to promote mental health prevention.

“Social media could be useful mainly for prevention.” UoS, F.

“If these digital applications have content about prevention, it can be helpful.” Unimib. F.

The main reason was the possibility of reaching a large number of people due to the wide diffusion and trends of digital tools.

“I completely agree with digital tools. It is the best way to speak about mental health for prevention, since nowadays most of all interactions among young people come from social media.” UoS, F.

Some students specified how a preventive intervention through digital tools could be useful, especially for people who found it difficult to directly seek support due to social anxiety.

“I think that an app could be more useful for prevention, in particular for people that suffer from social anxiety and have difficulty in searching for direct help.” UoS, M.

##### 3.2.5.3 Stigma Reduction

Only for Unimib students' opinion, digital tools could act as instruments to decrease the stigma around mental health. In more detail, digital content could promote the normalisation of symptoms through both psychoeducational content and virtual support by a professional.

“[Digital tools can be used] also to push on the fact that those who go to the psychologist are not crazy, but they are human, and this is useful.” Unimib, F.

Moreover, digital instruments could broaden general awareness about early symptoms and overcome stereotypes linked to mental health support.

“Maybe students will not approach counselling as a first tool because of the stigma. It could be different if they had the opportunity to understand what the early signs of mental health distress are and familiarise themselves with the environment, making it clear that it is not a judgmental environment. Digital tools can help in this.” Unimib, F.

Finally, digital tools could specifically address the needs of students who feel embarrassed about the possibility of developing psychological symptoms. The opportunity of being anonymously in contact with a professional through a digital screen is seen as a characteristic that could mitigate the feeling of shame.

“Maybe someone is ashamed to say that he goes to the psychologist. Digital tools are anonymous, and they can win the stigma around mental health.” Unimib, F.

“I think digital tools can be helpful for those who are afraid to search for face-to-face help and open up and believe that it is a negative thing.” Unimib, F.

### 3.3 Comparison between Unimib and UoS

As a whole, similarities in the responses from students at both universities were consistently found across the interviews in terms of content and themes. However, some differences emerged when comparing Italy and the UK. First, UoS students discussed their general perspectives on digital tools and mental health support more frequently than Italian students. Second, only students from the UK recognised psychotherapy or psychological support as useful content of digital tools, still acknowledging limited personalisation as a disadvantage of these instruments. Conversely, students from Unimib, but not from UoS, highlighted the potential of digital tools to reduce mental health stigma.

## 4 DISCUSSION

To our knowledge, this is the first cross-country, qualitative study on the role that digital tools could play in supporting the mental health of university students. Participants described the need for a multi-modal approach, including both digital tools and personal contact, as being key to the delivery of effective mental health support, in particular for treatment interventions. In addition, from students' perspective, social media, mobile apps, and podcasts could benefit from including some relevant mental health content, namely psychoeducational and psychological interventions, peer support, and specific interactive content. While wide availability and anonymity are perceived as advantages, lower efficacy compared to personal contact, lack of personalisation, and problematic engagement are emphasised as disadvantages of digital tools. Moreover, some specific potential roles for digital technologies emerged from the interviews, namely using these tools as an extension of standard treatment, as well as for prevention and reduction of mental health stigma. All these specific features enabled the definition of some students' needs about mental health, explaining through which potential mechanisms these could be partly met by digital tools. Our findings are largely consistent with previous research on students' needs and perspectives about the use of digital technologies for mental health support.

Interviewees expressed their need for accessible support since organisational issues, namely accessibility, proved to be among the key barriers to help-seeking among university students [13, 14, 52]. Thus, students from Unimib and Uos recommended social media, mobile apps, together with online platforms and podcasts, as useful ways to deliver mental health assistance, primarily due to their ready availability, defined as usability in a wide range of situations, circumstances, environments, and conditions [52]. Indeed, different categories of digital instruments are nowadays commonly used in information gathering and to pursue a steady social connection [17, 30]. In addition, the employment of digital tools in mental health support is taking hold among young people with promising effects [10, 20-23]. According to our sample, social media, primarily Instagram^®^, was the most suitable digital environment for the delivery of mental health content [53, 54]. As endorsed by previous evidence, the benefits of social media include wide diffusion among young people and an immediate communicative power, together with the opportunity to provide emotional connection and support [25, 27, 53, 55]. However, mainly thanks to their likelihood to guarantee anonymity, mobile apps and online platforms were also recognised as valid support in therapeutic practice and specific knowledge dissemination [56-58]. Similarly, podcasts, albeit recently introduced in common use [59], could be a reasonable instrument for circulating psychoeducational content [59, 60]. Furthermore, a broader use of social media by young students was reported during the COVID-19 pandemic [61, 62]. This was perceived both as an opportunity to remain connected and a potential risk factor for poor mental health [45, 63]. On the one hand, higher-education students often deem virtual networks and communities on social media platforms suitable for self-disclosure or help-seeking [29, 45, 64]. On the other hand, spending more time online was associated with self-isolation [45, 65], and the persistent comparison with peers through social content could lead to psychological distress [66, 67].

Moreover, students expressed their need for primary prevention and psychological support [13], suggesting the inclusion of useful content, namely psychoeducation, psychological interventions, peer support and specific interactive content, able to cover both primary, secondary, and tertiary preventive approaches. Consistently with recent findings, digital tools emerged as instruments suitable for discussing psychological issues, providing practical advice on how to search for help and offering both informative and scientific data to the general population [25, 26, 68-70]. In addition, interviewees emphasised peer support and mental health interactive content as interventions that could be offered to detect symptoms earlier or as first-line assistance. Peer support could be finalised through the sharing of personal experiences, coping strategies and advice [51, 53, 71], while interactive content could include tools to track emotion and mood as well as targeted activities and positive feedback [55, 69]. According to students from UoS, those who had already experienced a psychological condition could benefit from professional support or even from a structured psychotherapy treatment using digital devices [10, 20-23].

Consistent with previous evidence, another need that emerged from themes and sub-themes was the reduction of the stigma around mental health [72, 73]. In this study, students from Unimib specifically identified a role in stigma reduction for digital tools associated with the use of informative content shared with a wide audience via virtual platforms [74], as well as with the potential anonymity offered by these instruments [75]. More specifically, a good mental health literacy [76, 77], together with the opportunity to seek help without attracting attention, could reduce social anxiety and moderate the relationship between self-disclosure and embarrassment, recognised as a potential individual barrier to help-seeking in university students [13, 14, 75]. In addition, digital content could promote the normalisation of mental health symptoms, broadening general awareness about early symptoms and overcoming stereotypes linked to mental health support, thus reducing relevant stigma [73, 76,78].

Finally, the need for personal contact to manage psychological and mental health distress was emphasised across themes [32, 38, 79]. Although participants recognised the utility of digital tools in mental health support, human contact remained essential, even more so after the mandatory isolation and the excessive time online experienced during the COVID-19 pandemic [31, 45]. Thus, one of the disadvantages of digital tools identified by interviewees was their perceived lower efficacy due to the superiority of personal relationships compared to those mediated by a screen [35]. Participants justified their opinion by describing severe mental health issues as a “*real-world problem*” thus deserving a “*real-world solution*” [58]. Moreover, lower personalisation as compared to face-to-face approaches was highlighted as an additional disadvantage for online mental health support [35], with the main reason being the complexity of psychological conditions, which can be hardly managed through a digital platform [31]. Consistently, in students' opinion, digital interactions were less attractive than human contact, suggesting difficulties in engagement [19, 69]. Thus, increased personalisation and enhanced ease of engagement could be among the potential mechanisms defining how digital tools can meet students' mental health needs [35, 69]. Accordingly, students stated that digital instruments could be a good option as a starting way to familiarise themselves with mental health settings and further proceed with face-to-face treatment, thus highlighting the role of digital tools not as a replacement but as an extension of in-person therapy [79]. This confirms the expected greater effectiveness and adherence of interventions with an in-person element, either professional or peer, than fully automated or self-administered ones [79]. Thus, digital mental health support could be included in the context of multi-modal treatment with a greater benefit, at least for moderate psychological conditions [22, 58].

Despite most patterns being shared between the two countries, some differences emerged when comparing students from Italy and the UK. First, during the interviews, UoS students showed greater participation, more extensively describing their perspective and attitudes towards using digital tools for mental health support than students from Italy. Second, only students from the UK recognised psychotherapy or psychological support in general as useful content of digital tools, alongside the lack of personalisation as a relevant disadvantage. These findings could outline a greater awareness in UoS students about the use of digital technologies for mental health support, potentially related to specific sociodemographic characteristics and cultural factors [39-41]. In the UK, living on campuses or in shared residences is the most popular choice of accommodation for university applicants [80]. Despite several challenges being associated with moving from home into student accommodation [81], campus life could be an opportunity to enrich social experiences [1, 82] and peer support on information sharing, thus enacting discussion about different themes. This may produce a greater awareness and openness towards mental health-related issues [83-85]. Moreover, students from the UK could have a broader experience of mental health and related interventions due to national strategies for promoting university-based groups that lead conversation, culture change, and advocacy throughout campuses [43, 86-88]. Conversely, students based in Italy, except international and non-resident students, usually live with their parents [89, 90], thus experiencing both pros and cons of their living arrangements [91, 92]. It is possible this results in a more limited university social life experience and fewer peer group interactions than what is offered by a campus-based life, which may lead to a decreased opportunity to address mental health issues in the university setting. The higher demand for additional instruments to reduce stigma reported by students from Italy might be partly explained by their more limited opportunities to take part in the university's mental health advertisement and campaigns as compared with UK students more often living on campuses [87, 93, 94]. Indeed, students from Unimib, but not from UoS, clearly highlighted the reduction of mental health stigma as a key role of digital tools [1, 73, 74, 95].

### 4.1 Limitations

Our findings should be interpreted with caution, considering some methodological limitations [45]. First, although we attempted to diversify the sample to include a wide variety of students in terms of sociodemographic characteristics, male students were under-represented, thus limiting gender-based comparisons. Despite an appropriate distribution across age, degree programmes, and living conditions, interviewees were volunteers and might be students who were already well engaged with and interested in mental health topics. A relatively small number of participants had used digital interventions; therefore, most of the students' opinions reported were based on attitudes rather than experience. Second, the principal investigator's education and background might have influenced the interview content and subsequent interpretation through a possible dedicated focus on mental health issues. Nevertheless, interview questions were research-based and piloted before the start of the study to ensure understandability and face validity in relation to the topics covered, as well as maximum relevance. Moreover, the interviews were held in a private setting, which has been shown to minimise worries about privacy and anonymity, thus allowing participants to freely express themselves and take their time to explain their perspectives [96].

## CONCLUSION

The current study corroborated evidence on the potential utility of digital tools in the mental health support of university students. Benefiting from some specific features, namely accessibility and anonymity, and acting on potential disadvantages, digital solutions might meet some university students' specific needs. Since personal contact remains crucial to guarantee individual psychological well-being, these technologies should be integrated with face-to-face alternatives through a multi-modal approach. Further research is needed to provide additional insight into this field, considering also national and cultural barriers and facilitators to digital interventions.

## Figures and Tables

**Fig. (1):**
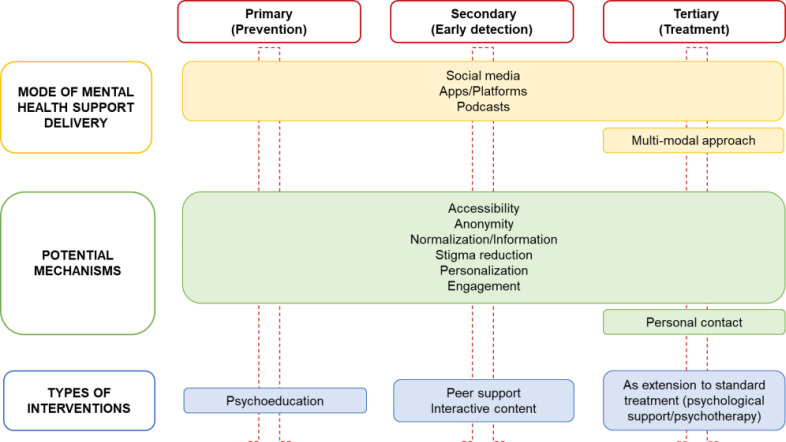
Thematic framework of students' perspectives on digital tools for mental health support.

**Table 1:** Sample characteristics.

**Characteristics**	**Total**	**Italy**	**UK**
-	N = 33 N (%) or Median (IQR)*	N = 15 (45%) N (%) or Median (IQR)*	N = 18 (55%) N (%) or Median (IQR)*
** Sex **			
Women	26 (79%)	12 (80%)	14 (78%)
Men	7 (21%)	3 (20%)	4 (22%)
****Age**, **yrs.****			
Median (IQR)*	22 (1.5)	22 (2)	21 (5)
** Nationality **			
Italian	15 (45%)	15 (100%)	0
British	13 (40%)	0	13 (72%)
Other	5 (15%)	0	5 (28%)
** Year of Study **			
First	7 (21%)	2 (13%)	5 (28%)
Second	12 (37%)	4 (27%)	8 (44%)
Third	5 (15%)	2 (13%)	3 (17%)
Fourth	6 (18%)	4 (27%)	2 (11%)
Fifth-Sixth	3 (9%)	3 (20%)	0
** Degree Programme **			
Applied/Formal Sciences^a^	4 (12%)	2 (13%)	3 (17%)
Economic/Legal Sciences^b^	6 (18%)	4 (27%)	2 (11%)
Medical Sciences^c^	9 (27%)	3 (20%)	6 (33%)
Natural Sciences^d^	6 (18%)	1 (7%)	4 (22%)
Psychosocial Sciences^e^	8 (24%)	5 (33%)	3 (17%)
** Accomodation **			
On Campus	8 (24%)	1 (7%)	7 (39%)
With family	11 (34%)	6 (40%)	5 (28%)
Alone	5 (15%)	3 (20%)	2 (11%)
Accommodation with roommates in university town	9 (27%)	5 (33%)	4 (22%)
** Awareness about Digital Mental Health **			
Yes	29 (88%)	11 (73%)	18 (100%)
No	4 (12%)	4 (27%)	0
** Digital Mental Health Users **			
Yes	6 (18%)	3 (20%)	3 (17%)
No	27 (82%)	12 (80%)	15 (83%)

**Notes:** *IQR: Interquartile range. ^a^Applied/Formal Sciences: Biomedical Engineering; Computer Sciences; Maths. ^b^Economic/Legal Sciences: Economics; Hospitality and Tourism; Law; Politics. ^c^Medical Sciences: Medicine and Surgery; Nursing; Nutrition; Paramedics; Veterinary Medicine. ^d^Natural Sciences: Biochemistry; Biology; Biomedical Sciences; Geology. ^e^Psychosocial Sciences: Education Sciences; Intercultual communication; Psychology; Sociology.

## Data Availability

The authors confirm that the data supporting the findings of this study are available within the article and its supplementary materials.
