# Supplementary material for: Understanding University Students' Perspectives towards Digital Tools for Mental Health Support: A Cross-country Study
Source: Clin Pract Epidemiol Ment Health. 2024 Feb 21;20:e17450179271467. doi: 10.2174/0117450179271467231231060255 (PMC11037510; doi:10.2174/0117450179271467231231060255)
Supplement: Supplementary file 1 — Supplementary material is available on the Publisher’s website. [file CPEMH-20-E17450179271467_SD1.pdf]

Clin. Pract. Epidemiol. Ment. Health
DOI: 10.2174/0117450179271467231231060255, 2024, 19, e17450179271467
ISSN: 1745-0179
1

SUPPLEMENTARY MATERIAL
OPEN ACCESS

Understanding University Students' Perspectives towards Digital Tools for Mental Health Support: A Cross-country Study

Ilaria Riboldi[1,*], Angela Calabrese[1], Susanna Piacenti[1], Chiara Alessandra Capogrosso[1], Susanna Lucini Paioni[1], Francesco Bartoli[1], Giuseppe Carrà[1,2], Jo Armes[3,#], Cath Taylor[3,#] and Cristina Crocamo[1,#]

[1]Department of Medicine and Surgery, University of Milano-Bicocca, Via Cadore 48, Monza 20900, Italy
[2]Division of Psychiatry, University College London, Maple House 149, London W1T 7BN, UK
[3]Faculty of Health and Medical Sciences, School of Health and Sciences, University of Surrey, Stag Hill, Guildford GU2 7XH, UK

© 2024 The Author(s). Published by Bentham Open.
This is an open access article distributed under the terms of the Creative Commons Attribution 4.0 International Public License (CC-BY 4.0), a copy of which is available at: https://creativecommons.org/licenses/by/4.0/legalcode. This license permits unrestricted use, distribution, and reproduction in any medium, provided the original author and source are credited.

Received: August 11, 2023
Revised: December 6, 2023
Accepted: December 20, 2023
Published: February 6, 2024

*Address correspondence to this author at the Department of Medicine and Surgery, University of Milano-Bicocca. Via Cadore 48, Monza 20900, Italy; Tel: +39 0257998647; E-mail: i.riboldi1@campus.unimib.it
#These authors equally contributed

Cite as: Riboldi I, Calabrese A, Piacenti S, Capogrosso C, Paioni S, Bartoli F, Carrà G, Armes J, Taylor C, Crocamo C. Understanding University Students' Perspectives towards Digital Tools for Mental Health Support: A Cross-country Study. Clin Pract Epidemiol Ment Health, 2024; 19: e17450179271467.
http://dx.doi.org/10.2174/0117450179271467231231060255

Send Orders for Reprints to
reprints@benthamscience.net

Supplementary S1. Interview Topic Guide

Introduction

Hello, my name is Ilaria, I am a PhD student at the University of Surrey, and I would like to invite you to participate in this research project, which is part of my PhD research.

The purpose of this study is: to assess and compare mental health among students from UniSurrey and the University of Milano Bicocca (UNIMIB, Italy), and to explore associated factors using a survey design; to assess mental health and well-being among UniSurrey and UNIMIB students during the COVID-19 outbreaks; to identify the areas of greatest interests and the main critical issues about students' psychological wellbeing for the design and evaluation of future preventive digital interventions.

This present stage of the study will involve interviewing university students about their perspectives and experiences of mental health and psychological well-being. The conceptual framework was developed starting from the existing literature on the field.

These interviews will help develop an online survey aimed at investigating different domains of mental health in university students.

In depth-interviews will be conducted by the principal investigator through an online platform (MS Teams) and will be recorded.

We will collect information on students' awareness and knowledge about university students' mental health and all the services offered to improve their well-being. Each interview will follow a semi-structured topic guide (see below), with no right or wrong answers, but in the form of a conversation.

Interviews will last between 20 to 30 minutes. Informed consent will be audio-recorded and checked verbally immediately prior to commencing the interview.

Participants will be asked for consent to have the interview audio and video recorded. Interviews will be transcribed for substantive content by the researcher, and they will be destroy once transcribed.

Participants will be informed they are free to discontinue an interview at any time until it concludes and may withdraw the data from that interview for up to seven calendar days following that interview by informing the researcher, without needing to give a reason.

Your personal data will be kept securely in accordance with data protection guidelines, and only be accessible to the immediate research team or responsible persons at the University.

Please could you confirm for me if you agree to participate in this study? Please could you confirm that you agree with the following points:

(read list from consent form)

Are you happy for me to record our interview? (If yes, start recording)

Opening questions

- Which degree programme are you enrolled in?
- What year are you in?
- How old are you?
- What is your nationality?
- Are you an international student?
- Can I ask you something about your accommodation?

University students and mental health/psychological well-being

- Do you think it is important to talk about mental health in university students?
- Before asking you more specific questions, can I start by asking how many people you think suffer from mental health problems at university? Are there specific subgroups of students that could be more at risk of mental health issues?
- In your opinion, what could be the causes of a low level of psychological well-being? Do you think it could be connected with academic worries, loneliness, change in habits, or other reasons?

University students and mental health's specific domains

- What do you think are the most common psychological/mental health problems faced by university students among depression, anxiety, stress, substance abuse, self-harm, suicidal ideation, eating disorders, poor sleep quality?
- Are there any other specific mental health problems you consider common among university students?
- In your opinion, what are the most common manifestations of a low level of psychological well-being?

Mental Health and Covid-19

- How do you think Covid-19 has influenced the students' psychological wellbeing?
- In your opinion, what are the main psychological problems related to the pandemic?

Intervention

- Prevention Programme And Centers For Wellbeing
  - In your opinion how often do university students seek help to improve their psychological difficulties?
  - What do you think can be done to improve students' mental health? In what ways could UniSurrey service for students' mental health be improved?
  - What do you know about prevention programmes offered by UniSurrey to improve students' mental health?
  - Are there any personal barriers, such as reluctance to seek help or stigma, which delay students accessing services?
  - Are there any service-related barriers, such as overbooking and limited staff, which delay students access to the service?

Areas to Explore:

- Referral route
- Waiting times
- Information sharing

Digital

- What do you know about digital interventions for the identification or treatment of psychological difficulties?
- Could a digital intervention be useful to prevent and treat low levels of psychological well-being in university students?
- Have you ever used one of these digital interventions (app, website)? If so, how did you find it?
- Which type of contents is it useful to include?

Concluding questions

- Are there any questions that we have not asked you that you were expecting? Why do you think this is important to ask about?
- If I have any further questions following on from today, would you mind if I contacted you by telephone or email to clarify?
- Would you like a copy of the final report when the study finishes? If so, how should we send this to you? (Get email/postal address and give them indication of when this might be).

Thank you very much for your time today.

Supplementary S2. Complete set of participants' quotes divided by themes and sub-themes.

Themes and Sub-themes	Quotes
**PREFERRED MODE OF MENTAL HEALTH SUPPORT DELIVERY***	
**Social media****	● Digital tools could be really useful for mental health support. It is the best way to reach students. I am a students' representative on campus, and I know that most of all interaction comes from social media, for example, Instagram. And there are a lot of people just sit down going through Instagram Stories. And then once appetizing things pop-up they are actually quite likely to take a look to them. And so, I definitely think that Instagram Stories are the best way to go [for mental health support]. UoS, F.

-	● Let's say online stuff, especially social media like Facebook, just give to young people an insight that they're not alone nor judged. UoS, F.<br>● There is an increase of people on social media with real mental health problems talking about their experiences. It helps you to feel understood. UoS, F.<br>● Probably Instagram is the best social media to use for mental health support because that's what most students use, and it is very direct and easy to access. UoS, F.<br>● You know it's like everything is on social media and it makes so much easier to reach out to people, especially the youngest ones. UoS, F.<br>● Maybe it would help some social media, some sponsorship, some mini pills, to help bring students closer to mental health. For me it is a very useful way to reach out all that part of kids of my generation who still have that kind of distrust and low awareness about mental health. Unimib, F.<br>● Instagram is one of the most common social networks among young people, so it's also the tool to get closer to us and speak about mental health. Unimib, F.<br>● Ninety percent of people use Instagram, especially my age, and it can give information about mental health. Unimib, F.<br>● We are always connected with others on Instagram, and we can share our experience and emotions in every moment. Unimib, F.<br>● I know about some Instagram pages already offering content and articles about mental health. Unimib, F.<br>● Surely the social network has a greater effect [on mental health support] than apps, because they are more widespread, and apps are a bit backward. Unimib, F.
**Mobile apps/Online platforms****	● An online platform where people can anonymously talk about their mental health [could be useful]. UoS, F.<br>● An app on your phone could be very useful to give information and promote mental health. UoS, F.<br>● I am thinking about an app where people can do some type of psychotherapy or mindfulness. UoS, F.<br>● Mobile apps could be useful for mental health support. On apps people, young people or anyone can just anonymously say what they're going through, and then they can get some professionals who can help them without any charge. UoS, F.<br>● On apps people, can just anonymously say what they're going through. UoS, F.<br>● I know there is a very useful app to help students manage their self-harm. UoS, F.<br>● It could be useful to create an app to connect you with your tutors or the university's Centre of Wellbeing in a more fluid way to receive psychological support. UoS, M.<br>● I think that an app could be more useful for prevention in particular for people that suffer from social anxiety, because it is easier to search for help behind a screen. It can play right into whatever your problem is at the moment. I believe that an app would be much more useful to students. UoS, M.<br>● I think that an app could be more useful for prevention in particular for people that suffer from social anxiety. UoS, M.<br>● Websites or blogs can provide information on mental health support. Unimib, F.<br>● Apps could be beneficial [for mental health support]. They help to get a little closer to people as first aid. Unimib, F.<br>● Students are made aware of many issues through almost exclusively the use of IT means, social media, online pages. Unimib, F.
**Podcasts****	● Listening to podcasts about mental health could be very useful. UoS, F.<br>● Podcasts about mental health helped me a lot. They didn't get change my mood, but they felt like reassuring as I'm not the only one. UoS, F.<br>● Podcasts talking about mental health helped me, increasing my personal knowledge on mental health, so I think it could be good to create podcasts on mental health. UoS, F.<br>● I would like to say that I personally would be interested in face-to face meetings or online webinars on mental health. Otherwise, I would like to listen to something like a podcast on the topic. Unimib, F.
**USEFUL CONTENT***	
**Psychoeducation/Information on mental health****	● In my opinion the most useful content could be something about self-awareness and self-love, that are really important for mental health. UoS, F.<br>● Maybe something about prevention and information on mental health could be done through digital tools. I completely agree with digital tools. It is the best way to speak about mental health. UoS, F.<br>● I think motivational quotes could be useful to increase awareness about mental health. UoS, F.<br>● I propose a mobile app, where you can login and find information about mental health. UoS, M.<br>● ...just general advertisement on mental health trough apps. UoS, F.<br>● ...a university account, with information on mental health. University would need to advertise it in order to reach as many people as possible. UoS, F.<br>● ...you know it's like everything is on social media and it is so much easier to reach out to people using digital tools to circulate information on mental health. UoS, F.<br>● They [digital tools] can be useful also to get a little closer to people and disseminate information about mental health. Unimib, F.<br>● If these digital applications have content about prevention and information on mental health it can also be helpful. Unimib, F.<br>● I personally have obtained a lot of information on mental health online. Unimib, F.<br>● On Instagram I follow many psychologists who do a lot of awareness about mental health in a professional way. Unimib, F .<br>● You can use time to read sentences on social media, not only motivational, but with scientific data on mental health. Unimib, F.<br>● Digital tools can allow you to know how to search for a psychologist. Unimib, F.

**Psychological support/Psychotherapy****	● In my opinion it could be useful to have a platform where people can anonymously talk about their mental health and receive psychological support. UoS, F.<br>● ...an app where people can receive some type of psychotherapy. UoS, F.<br>● I'm probably not the right generation, I guess I don't have any personal experience because I don't rely on digital communication myself. However, I do know apps like “calm” for mental health. I know I am now at university, but I used to work in a school and self-harm was quite prevalent and there was an app to help students manage their self-harm and was really successful. UoS, F.<br>● It could be useful to create an app to connect you with your tutors or the university’s Centre of Wellbeing in a more fluid way to receive general support. UoS, M.<br>● There is this app where people, young people or anyone, can just anonymously say what they're going through, and then we can get some professionals who can help them without any charge, 'cause going directly to a professional is extremely expensive. If you want to have a session or anything is extremely costly. UoS, F.<br>● I am thinking about something simple with a space where you can ask questions and [receive] support from someone who is a real person. UoS, M.
**Peer support****	● I feel like social media could be a way of sharing personal stories and it could be really good for peer support on mental health. UoS, F.<br>● There is an increase of people talking about their experiences with mental health problems. It could be useful for advice and real coping mechanisms. UoS, F.<br>● I feel like social media and apps really have their pros and cons because a lot of people find a way of healing in social media by sharing their stories to other people and it could be really good for peer support on mental health. UoS, F.<br>● Let's say online help is useful, on Facebook or through podcasts, anything that'll just give people an insight that they're not alone. They felt reassuring, like you are not the only one. There are so many people going through even worse situations. Everyone is there to help them without judging them at all. UoS, F.<br>● I propose to create and share videos, interviews, experiences, personal testimonials precisely referred to the aspect of the student's mental health. Unimib, F.
**Specific interactive content****	● I propose an app which gives people small activities to do throughout the day and a tracking of their emotion. At the end of the day, they have to report back what they did and how they feel, and they receive some advice. UoS, F.<br>● Something interactive about mental health. With interaction it is easier for people to use an app or follow a social media account. UoS, F.<br>● Digital interventions need to have interactive content, like a tracking of the emotions to detect early mental health problem. Unimib, M.
**PERCEIVED ADVANTAGES OF DIGITAL TOOLS***	
**Availability****	● Booking an appointment to go physically to a psychological session is harder than just using a digital platform, that is much more accessible. UoS, F.<br>● I completely agree with digital tools. It is the best way to speak about mental health, since nowadays most of all interaction among young people comes from social media, for example, Instagram. UoS, F.<br>● I think digital tools help, especially with students. I think accessibility is very important, as students are, on the whole, quite lazy. UoS, M.<br>● In my opinion [the use of digital tools for mental health support] is a really, really good idea. I think it could help people, especially because everything is online now. I think a lot of people would probably feel comfortable seeking help online because it is easily accessible. UoS, F.<br>● I think it is really good [to use digital tools for mental health] since so many people use their phones and they are always online. UoS, F.<br>● It makes easy access for 24 hours a day and it makes it anonymous. UoS, F.<br>● ...you know it's like everything is on social media and it is so much easier to reach out to people using digital tools, and also to circulate information on mental health. UoS, F.<br>● Probably Instagram is the best social media to use for mental health support because that's what most students use. UoS, F.<br>● In my opinion a digital contact is essential because today obviously students, but not only, in any case all young people, become aware of many topics through almost exclusively the use of Instagram, social media in general, online pages, etc. Unimib, F.<br>● Instagram is one of the most common social networks among young people, so it's also a tool to get closer to us and speak about mental health. Unimib, F.<br>● Ninety percent of people use Instagram, especially my age, and it can give information about mental health. Unimib, F.<br>● Instagram and social media in general are direct and easy to access for students of my generation. We are always connected. Maybe it could be useful to create some content on social media for communication on mental health. Unimib, F.
**Anonymity****	● I think digital tools can be useful because interactions can be kind of anonymous. So, it is easier to reach out or start reaching out and finding the right support without getting noticed. UoS, F.<br>● Mobile apps could be useful for mental health support. On apps people, young people or anyone can just anonymously say what they're going through, and then they can get some professionals who can help them without any charge. UoS, F.<br>● Some people, including me, may not want to go and speak to someone face-to-face because it is a bit stressful, and it's harder than speaking over the computer or something digital. UoS, F.<br>● It makes easy access for 24 hours a day and it makes it anonymous. UoS, F.<br>● I know for a lot of people it is so much easier to open up in front of a digital platform for mental health support, because no one knows them. UoS, F.<br>● I think making a phone call to a helpline is much more difficult for students than using an app and doing things behind the screen. They can have some social anxiety and prefer to be anonymous. UoS, M.<br>● An online platform where people can anonymously talk about their mental health [could be useful]. UoS, F.<br>● Maybe someone is embarrassed to say that he has symptoms. Digital tools could be useful because they are anonymous. Unimib, F.

PERCEIVED DISADVANTAGES OF DIGITAL TOOLS*	
**Lower efficacy than personal contact****	● I think they [digital tools] can only be used together with, not instead of, personal contact. I prefer personal contact. UoS, F.<br>● I think it just helps to talk to someone about mental health and not to use digital instruments. UoS, F.<br>● Mental health issues are a real-world problem, and you have to address it in the real world. I think that you can simply search for National Health Service. I don’t trust internet stuff. UoS, M.<br>● I don't find it helpful like just going on social media, because I'd rather talk to someone about my psychological conditions. UoS, F.<br>● When you have a serious mental health problem it is better to go to speak to someone face-to-face. UoS, M.<br>● I felt more the stressful aspect of social media instead of the beneficial one. Unhealthy competition and comparison with others. Personal contacts are better. UoS, M.<br>● I would honestly see [digital tools] as less attractive than human contact, especially at the beginning. Unimib, F.<br>● Honestly [digital for mental health] is much less effective and attracts much less than an in-person one-to-one meeting. Unimib, F.<br>● Digital doesn't make much sense. I don't think it could be useful for mental health, because in my opinion human relationships are essential. Physical contact has a completely different impact on mental health, and I realised it during the pandemic. Online stuff can be useful but not for teaching nor for mental health support. Unimib, M.
**Lack of personalisation****	● Mental health is such a broad term of things. It is impossible to create digital content that can be suitable for everyone. UoS, F.<br>● Sometimes [digital tools] cannot be specific enough for all type of mental health problems. UoS, M.<br>● For university students a digital intervention needs to be more personalised than the ones that you can find nowadays. UoS, M.
**Problematic engagement****	● I think the main sort of thing to worry about in the use of digital tools for mental health support is how to get people to use them over time. UoS, F.<br>● I would honestly see [digital tools] as less attractive than human contact. Users need to see that instead digital applications could be interesting to continue to use them. Unimib, F.<br>● Apps are, in my opinion, a bit backward. I don't know how much the apps would inform or even prevent mental health issues, creating a habit of using them. Unimib, F.
**PUTATIVE ROLES IN MENTAL HEALTH SUPPORT***	
**Complement/extension of standard treatment****	● Digital platforms are very useful as a starting point for mental health support. Then you can go on together with a face-to-face psychotherapy. UoS, F.<br>● I think they [digital tools] can only be used together with, not instead of personal contact. UoS, F.<br>● [Digital tools] can help the progression to meeting a mental health professional or counsellor and make the initiation of therapy easier. UoS, F.<br>● I think [digital tools] would be part of a multi-pronged programme, also including personal contact. UoS, F.<br>● I think digital utility depends on the severity of the mental health problem. It is really useful for initial situations. UoS, M.<br>● Digital interventions tend to be about mindfulness, and I know that it's good for general mental health, but someone who's depressed, maybe doesn’t have desire to do mindfulness. So, they may be more useful to start approaching mental health support. UoS, F.<br>● I know apps about mental health, and I think they could be a first aid in mental health support. Unimib, F.<br>● I do not believe so much in digital tools as a solely support for mental health. Maybe they can be of help during a therapy. Unimib, F.<br>● For students suffering from a very superficial and initial problem, they [digital tools] can also be helpful, but not as the unique treatment. Unimib, F.
**Prevention****	● Social media could be useful mainly for prevention. UoS, F.<br>● I think they're [digital tools] better as like a preventative intervention for mental health. UoS, F.<br>● I completely agree with digital tools. It is the best way to speak about mental health for prevention, since nowadays most of all interactions among young people come from social media. UoS, F.<br>● I completely agree with digital tools. It is the best way to speak about mental health, since nowadays most of all interactions among young people come from social media. UoS, F.<br>● There is an increase of people with real mental health problems talking about their real experiences through social media. It could be useful for advice, prevention and real coping mechanisms and prevention. UoS, F.<br>● If it is preventive it can also be helpful. Unimib, F.<br>● If these digital applications have content about prevention it can be helpful. Unimib, F.<br>● They [digital tools] can be useful also to get a little closer to people and circulate information about mental health, to increase prevention. Unimib, F.
**Stigma reduction****	● Maybe someone is ashamed to say that he goes to the psychologist. Digital tools are anonymous, and they can win the stigma around mental health. Unimib, F.<br>● Maybe students will not approach counselling as a first tool because of the stigma. It could be different if they had the opportunity to understand what the early signs of mental health distress are, familiarize themselves with the environment, making it clear that it is not a judgmental environment. Digital tools can help in this. Unimib, F.<br>● I think digital tools can be helpful for those who are afraid to search for face-to-face help and open up and believe that it is a negative thing. Unimib, F.<br>● For me they [digital tools] are a very useful way to involve all that part of young people of my generation who still have that kind of distrust about mental health. I myself, are participating with a group of psychologists to slightly increase the knowledge that exists with respect to false stereotypes about the figure of the psychologist or psychotherapy; we are trying to make it clear that psychology is not just Freud and a couch, which unfortunately is still the image linked to psychology. I am trying to create multimedia content made by students for students. Just to break down the initial wall I think social media are very important. In the past when there have been such interventions there have been benefits. Unimib, F.

-	● [Digital tools can be used] also to push on the fact that those who go to the psychologist are not crazy, but they are human, and this is useful. Unimib, F.

*Themes
**Sub-themes
